# Translation, cultural adaptation, and validation of the Brazilian Portuguese version of the Higher Education Stress Inventory (HESI-Br)

**DOI:** 10.47626/2237-6089-2021-0445

**Published:** 2023-09-22

**Authors:** João Pedro Gonçalves Pacheco, Maurício Scopel Hoffmann, Luiza Elizabete Braun, Isabella Poletto Medeiros, Damaris Casarotto, Simone Hauck, Fabio Porru, Michael Herlo, Vitor Crestani Calegaro

**Affiliations:** 1 Programa de Pós-Graduação em Ciências da Saúde Universidade Federal de Santa Maria Santa Maria RS Brazil Programa de Pós-Graduação em Ciências da Saúde, Universidade Federal de Santa Maria (UFSM), Santa Maria, RS, Brazil.; 2 Programa de Residência em Psiquiatria UFSM Santa Maria RS Brazil Programa de Residência em Psiquiatria, UFSM, Santa Maria, RS, Brazil.; 3 Departamento de Neuropsiquiatria UFSM Santa Maria RS Brazil Departamento de Neuropsiquiatria, UFSM, Santa Maria, RS, Brazil.; 4 Programa de Pós-Graduação em Psiquiatria e Ciências do Comportamento Universidade Federal do Rio Grande do Sul Porto Alegre RS Brazil Programa de Pós-Graduação em Psiquiatria e Ciências do Comportamento, Universidade Federal do Rio Grande do Sul (UFRGS), Porto Alegre, RS, Brazil.; 5 are Policy and Evaluation Centre London School of Economics and Political Science London United Kingdom Care Policy and Evaluation Centre, London School of Economics and Political Science, London, United Kingdom.; 6 Faculdade de Medicina UFSM Santa Maria RS Brazil Faculdade de Medicina, UFSM, Santa Maria, RS, Brazil.; 7 Faculdade de Psicologia UFSM Santa Maria RS Brazil Faculdade de Psicologia, UFSM, Santa Maria, RS, Brazil.; 8 UFSM Santa Maria RS Brazil Coordenadoria de Ações Educacionais, UFSM, Santa Maria, RS, Brazil.; 9 Laboratório de Pesquisa em Psiquiatria Psicodinâmica Hospital de Clínicas de Porto Alegre Porto Alegre RS Brazil Laboratório de Pesquisa em Psiquiatria Psicodinâmica, Hospital de Clínicas de Porto Alegre, Porto Alegre, RS, Brazil.; 10 Department of Public Health Erasmus University Medical Center Rotterdam The Netherlands Department of Public Health, Erasmus University Medical Center, Rotterdam, The Netherlands.

**Keywords:** Psychological stress, university, education, psychometrics, factor analysis

## Abstract

**Objectives:**

There are no validated instruments to measure education-related stress in Brazilian university students. Thus, we aimed to translate and test the internal reliability, convergent/discriminant validity, and measurement equivalence of the Higher Education Stress Inventory (HESI).

**Methods:**

The translation protocol was carried out by two independent translators. The instrument was culturally adapted after a pilot version was administered to 36 university students. The final version (HESI-Br) was administered to 1,021 university students (mean age = 28.3, standard deviation [SD] = 9.6, 76.7% female) via an online survey that lasted from September 1 to October 15, 2020. The factor structure was estimated using exploratory factor analysis (EFA) on the first half of the dataset. We tested the best EFA-derived model with confirmatory factor analysis (CFA) on the second half. Convergent/discriminant validity was tested using the Depression, Anxiety and Stress Scale (DASS-21). Sex, age groups, period of study, family income and area of study were used to test measurement equivalence.

**Results:**

EFA suggested five factors: career dissatisfaction; faculty shortcomings; high workload; financial concerns; and toxic learning environment. CFA supported the five-factor model (15 items), but not a higher order factor, suggesting multidimensionality. All five factors presented acceptable internal reliabilities, with Cronbach’s α ≥ 0.72 and McDonald’s ω ≥ 0.64. CFA models indicated that the HESI-Br and DASS-21 assess different but correlated underlying latent constructs, supporting discriminant validity. Equivalence was ascertained for all tested groups.

**Conclusion:**

The 15-item HESI-Br is a reliable and invariant multidimensional instrument for assessing relevant stressors among university students in Brazil.

## Introduction

Psychological stress is high among university students globally and prevalence estimates can reach 99.2%.^1-5^ While a moderate level of psychological stress may increase individuals’ resilience,^6^ exposure to a high level of stress is associated with mental health problems (e.g., insomnia, depression, anxiety, and burnout)^5,7-9^ and worse academic outcomes (e.g., lower grade point average [GPA] and higher dropout rates).^10-12^ Moreover, multiple factors are associated with psychological stress among students, including academic overload,^5,13^ uncertainty and insecurity about the future,^13^ low income,^2^ and lack of self-esteem and motivation.^5^ However, there are still few scales validated for screening students under high levels of psychological stress related to the higher education setting.

The Higher Education Stress Inventory (HESI)^7^ was developed in 2005 aiming to provide a reliable tool to measure stress in higher education level students.^7^ Originally inspired by the Perceived Medical School Stress (PMSS) instrument,^14^ the scale aims to measure the presence of psychological stress in settings other than medical schools. As such, it was constructed to capture many of the stressors that students are exposed to in higher education, such as those mentioned above (e.g., academic overload, etc.). The HESI has previously been used to assess stress levels among Swedish^7^ and Korean^8^ medical students, Jordanian nursing students,^15^ Ugandan university students,^16^ and physicians in their first postgraduate year.^17^ Currently, the scale is validated for Arabic^15^ and Korean populations.^8^ Total HESI score has been associated with depressive symptoms,^7,8^ which have estimated pooled prevalence rates that vary from 24.4 to 42.6% among university students.^18-21^

In Brazil, the rate of university enrollment increased by 283.4% over the past 20 years. With more than 8.6 million people^22,23^ in higher educational settings, there is a need to assess stress among Brazilian university students. Additionally, students living in low and middle-income countries, such as Brazil, are affected by additional socioeconomic factors, such as lower income and higher discrimination.^24^ These factors might ultimately result in higher stress in university students.^2,3,25^

To our knowledge, no instruments focusing on measuring academic stress among university students have been validated for the Brazilian population. Thus, our research aimed to 1) translate the HESI scale into Brazilian Portuguese, 2) culturally adapt it, 3) test its structure and internal reliability, 4) test its convergent/discriminant validity, and 5) test its measurement equivalence across groups selected by different characteristics in a large sample of university students from Brazil.

## Methods

### Recruitment and data collection

Development of the Brazilian Portuguese version of the HESI (HESI-Br) is part of the COVIDPsiq study, which is a longitudinal survey of mental health in the context of the coronavirus disease 2019 (COVID-19) pandemic. Full details can be found elsewhere.^26^ Briefly, COVIDPsiq aimed to follow-up post-traumatic, depressive, and anxiety symptoms in Brazilians during the COVID-19 pandemic. The survey was conducted from April 2020 to February 2021 in four waves of assessment, using a non-probabilistic convenience sample. The study was publicized through social media platforms, a corporate mailing list, and digital and press media. Data were collected using the SurveyMonkey online platform. The choice of an electronic survey was based on the possibility of reaching more participants while respecting social isolation restrictions in Brazil. The research was approved by the human research ethics committee at the Universidade Federal de Santa Maria (CAAE: 30420620.5.0000.5346).

### Participants

The criteria for participation in the study to validate the scale were: (a) being a native Brazilian or residing in Brazil; (b) being over 18 years of age; (c) having access to digital equipment; (d) being literate; and (e) being a university student (at any level, e.g., undergraduate, graduate, postgraduate). All individuals participated voluntarily and provided informed consent online. In total, the survey period covered approximately 11 months. The questionnaire for each phase remained available for 1 month on average. The third phase, in which the HESI-Br was administered, extended from September 1 to October 15, 2020. A total of 2,303 respondents participated in the third phase of the larger longitudinal study. Of these, 1,021 were university students and answered the HESI-Br questions. Sociodemographic data for the sample are shown in Table 1.

**Table 1:** - University students’ sociodemographic characteristics

Students’ characteristics	Sample (n = 1,021)
Age, mean (SD), years	28.3 (9.6)
Missing, n	3
Gender, n (%)	
Male	235 (23.0)
Female	783 (76.7)
Missing	4 (0.3)
Family income, n (%) (BRL)	
Low (0 to 2,004)	223 (21.8)
Middle (2,005 to 8,640)	502 (49.2)
High (8,641+)	292 (28.6)
Study level, n (%)	
Bachelor	619 (60.6)
Residency, specialization	132 (12.9)
Masters, doctorate, or post-doctoral positions	267 (26.2)
Missing	3 (0.3)
Area of study, n (%)	
Technology and exact sciences	250 (24.5)
Health-related sciences	379 (37.1)
Social sciences, education, and arts	380 (37.2)
Missing	12 (1.2)
DASS-21 scores, mean (SD)	
Depression subscale	13.7 (11.3)
Anxiety subscale	10.0 (9.7)
Stress subscale	17.2 (10.7)

BRL = Brazilian Real (currency unit); DASS-21: Depression, Anxiety and Stress Scale; SD = standard deviation.

### Measures

#### Depression, Anxiety and Stress Scale (DASS-21)

Based on the tripartite model of depression and anxiety, the DASS-21 is a short version derived from the DASS-42, both developed by Lovibond and Lovibond.^27^ It is an instrument to measure symptomatology in three domains (depression, anxiety, and stress). It has 21 items with a four-point Likert response scale (0 = strongly disagree; 3 = strongly agree). The DASS-21 was translated into Brazilian Portuguese and validated in patients from two hospitals in Southern Brazil.^28^ A recent study^29^ examined the psychometric properties of the DASS-21 in eight countries, including Brazil, suggesting that the DASS-21 is best represented with a general distress factor. A second-order model had acceptable fit according to Zanon et al.,^29^ and was used to test convergent/discriminant validity of the HESI-Br.

#### Higher Education Stress Inventory (HESI) – Original version

Originally inspired by the PMSS,^14^ the HESI aims to assess the presence of educational stress in university students.^7^ It is a 33-item self-report instrument that uses a four-point Likert response scale ranging from 1 (does not apply at all) to 4 (applies perfectly). Ten items are reversed because they indicate absence of stress. Therefore, higher scores indicate higher educational stress levels.

The original HESI factor analysis identified a model comprising 24 items loading on seven factors. The factors also presented low to acceptable α values and were identified as: (I) Worries about future competence (α = 0.78); (II) Non-supportive climate (α = 0.71); (III) Faculty shortcomings (α = 0.69); (IV) Workload (α = 0.62); (V) Insufficient feedback (α = 0.65); (VI) Lack of commitment (α = 0.62); and (VII) Financial concerns (α = 0.59).^7^

## Translation and cross-cultural adaptation

Translation and cross-cultural adaptation were conducted in eight steps, according to the ISPOR Guidelines^30^ and with permission from the original author. Permission to publish the final instrument in its entirety was also obtained from the original author. Translation from English to Brazilian Portuguese was performed by two independent Brazilian professionals, both specialized in psychiatry and fluent in English. (1) Initially, the original HESI was translated into Portuguese by the first and second translator. (2) After comparison of the two translations, they produced a consensus version. (3) This version was sent to a third psychiatrist, with extensive knowledge of English, who evaluated and improved the translated version. (4) Next, the HESI-Br was back-translated into English by a professional translator and compared to the original version by the translators from step 1. (5) The Brazilian version was then adapted according to the differences found in the back-translation. (6) Cross-cultural adaptation was performed using a pilot version of the scale, to which 36 students responded via Google Forms and were asked to comment on any difficulties they had with specific items. Subsequently, a video conference was held with nine undergraduate students who are part of the COVIDPsiq project, for further information on how to improve comprehensibility (cognitive interview). A final culturally adapted version, the HESI-Br (online-only Supplementary Material S1), was administered to university students who participated in the third phase of the large longitudinal study. A flowchart illustrating the process is available in Figure S1, available as online-only supplementary material.

## Statistical analyses

First, 10 positive-oriented items of the translated version of the HESI scale (Q2, Q6, Q8, Q10, Q13, Q17, Q19, Q26, Q27, and Q33) were reverse-coded. The frequencies of responses per item are shown in Table S1, available as online-only supplementary material. Second, EFA and CFA were performed on randomly split halves of the dataset. Item response theory (IRT) analyses were performed on the CFA sample. Analyses of measurement equivalence and convergent/discriminant validity with DASS-21 were conducted on the whole dataset. A flowchart illustrating the data analysis plan is provided in Figure S2.

### Exploratory factor analysis (EFA)

We used the first half of the dataset (n = 511) for the EFA. The Kaiser-Meyer-Olkin (KMO) statistic was calculated to verify the data’s sampling adequacy for analysis and the result was evaluated according to Kaiser.^31^ A KMO cutoff of 0.5 was considered for the acceptability of individual items. Bartlett’s test of sphericity^32^ was used to evaluate whether correlations between items were sufficiently large for factor analysis. A parallel analysis was conducted using weighted least squares as factoring method (a scree plot is shown in Figure S3).

EFA was conducted on a polychoric matrix of the 33 items with oblimin rotation. The best structure was selected based on the following criteria: (a) items with fewer cross-loadings; (b) items with factor loadings > 0.3; and (c) factors with at least three items per factor. The best model was further filtered to keep the number of items per factor equal across the factors, based on the items with the highest factor loadings.

### Confirmatory factor analysis (CFA)

The CFA was conducted using the second half of the dataset (n = 510). It was carried out using delta parameterization and weighted least squares with diagonal weighted least square mean and variance adjusted (WLSMV) estimators. Global model fit was evaluated with root mean square error of approximation (RMSEA), comparative fit index (CFI), Tucker-Lewis index (TLI), and standardized root mean-square residual (SRMR) indices. RMSEA values lower than 0.060 and CFI or TLI values higher than 0.950 indicate a good-to-excellent model.^33^ An SRMR less than or equal to 0.100 indicates adequate fit, and values less than 0.060 in combination with previous indices indicate good fit.^33^ Using the EFA-derived model, we tested whether a correlated or a second-order version of the model better represents the HESI factor structure. A χ^2^ test was performed to test the difference between models. Factor reliability was examined using Cronbach’s α^34^ and McDonald’s ω.^35^

### Multidimensional item response theory (IRT) analysis

Item information (IIC) and item characteristic (ICC) curves were generated using the Graded Response Model for polytomous analysis and quasi-Monte Carlo expectation maximization (QMCEM) as estimation algorithm. These curves are based on the two-parameter IRT model, comprising parameter α (item discrimination) and parameter β (item difficulty).

Parameter α represents the rate at which the probability of answering a response category changes, given the construct level. It is the slope of the item characteristic curve, which is constant for all categories of the same item. Item discrimination helps to differentiate individuals with similar levels of the latent construct because it marks where, in the latent construct, the probability of answering items increases. Parameter β indicates the 50% probability of endorsing a given category or higher in the latent construct (i.e., τ thresholds) for each HESI-Br item (e.g., from “totally disagree” to “somewhat disagree”). It therefore informs the construct level that is necessary to change from one category to another. Parameter β is calculated by τ/λ, where λ is the standardized factor loading of a given item.

The IIC is calculated by multiplying the probability of answering a response category by the probability of not answering it, which is represented along the y-axis. The apex of the information curve is where parameter β is located (x-axis). The IIC illustrates the capability of each HESI-Br item to inform on the latent construct of academic stress and can discriminate those items that are more important to capture the information. The ICC depicts parameter α on the slopes of each response category curve, the probability of endorsing a given category (y-axis), and parameter β (x-axis). The IIC and ICC are relevant because, for example, an item may inform little at the lower end of the distribution of a given construct and might therefore work better to discriminate individuals at the upper end of the construct distribution, rather than those at the lower end (i.e., it can better discriminate people with higher rather than lower stress levels).

### Measurement equivalence

Measurement equivalence testing allows us to understand whether the mean score differences of a given test/questionnaire across different groups are due to true differences in the mean levels of the latent construct. In other words, it provides information on whether score differences are solely given by changes in the latent construct and not by exogenous sources of variation.

ME testing was carried out for groups selected by sex, age (18 to 25 years; 26 to 39 years; 40+ years), study level (bachelor; residency or specialization; masters, PhD or post-doc), family income (BRL 0 to 2,004; BRL 2,005 to 8,640; BRL 8,641+), and area of study (exact sciences or technology; health sciences; social sciences, education or arts). These tests were conducted using the whole dataset. Missing data were handled with pairwise deletion, since for this part of the analysis some sociodemographic variables had missing values (n missing for gender = 4; for study level = 3; for area of study = 12; for age = 3).

ME was tested by using multigroup CFA (MG-CFA) using the Wu and Estabrook approach.^36^ It consists of applying a sequence of constraints and comparing global model fit indices between each constrained model. The first step is to establish configural equivalence by constraining the model factor structure to be the same across groups. The second step is to establish threshold equivalence by further constraining item thresholds to be the same across groups. The third step is to establish metric equivalence by further constraining item factor loadings to be the same across groups (i.e., an increase of one unit on the scale has the same meaning across the compared groups). The fourth step is to constrain latent intercepts to be equal to establish scalar equivalence (i.e., respondents from different groups with the same value on the latent factor would have the same score on the observed indicators). Achieving scalar equivalence means that the questionnaire’s scores are comparable between groups. Thus, we tested whether the HESI-Br models in each group are structurally similar (configural equivalence), whether items are informing symptoms at equivalent level (threshold equivalence), whether they are equally correlated with the latent factors (metric equivalence), and whether latent means are equivalent (scalar equivalence). Values of ΔCFI < 0.01 and ΔRMSEA < 0.015 or ΔSRMR < 0.010 between nested models with increasing levels of constraints indicate equivalence.^37-39^

### Convergent/discriminant validity

CFA models including the HESI-Br and DASS (second order model) were used to test whether the two scales assess the same underlying latent construct (convergent validity) or if they inform on two correlated, but separate constructs (discriminant validity). We fitted a second-order model, where the HESI-Br (five factors) and DASS-21 (three factors) loaded on a higher-order factor (i.e., testing convergence by modeling the correlation between HESI-Br and DASS-21 as originating from the same source/latent factor) and a two-correlated factor model in which DASS-21 was modeled as a second-order model (“internalizing symptoms”) and HESI-Br factors were allowed to correlate with the DASS-21 higher-order factor (i.e., testing discriminant validity by modeling HESI-Br and DASS-21 as independent constructs, while allowing them to correlate). Fit indices (RMSEA, CFI, TLI, and SRMR) were compared between models. A χ^2^ test was performed to test the difference between models.

Measurement equivalence was analyzed using the measEq.syntax function in the “lavaan” package in R.^40^ CFA and convergent/discriminant validity analyses were carried out using the lavaan package in R.^40^ IRT analysis was carried out using the “mirt” package in R.^41^ R version 4.1.0 was used for all analyses (The R Foundation for Statistical Computing 2021).

## Results

### Exploratory factor analysis (EFA)

The Bartlett test p-value was 0, indicating that correlations between items were sufficiently large. The KMO value was = 0.85, indicating that the sample size was good. All KMO values for individual items were also acceptable (> 0.72). Parallel analysis suggested eight factors. Table 2 shows the factor loadings after rotation. Five factors had at least three items with factor loadings > 0.3 without any cross-loadings. Thus, we retained 15 items and five factors for the CFA. After examining the item content of each factor and inspired by the original instrument, we named factor 1 as career dissatisfaction, factor 2 as faculty shortcomings, factor 3 as excessive workload, factor 4 as financial concerns, and factor 6 as a toxic learning environment.

**Table 2:** Higher Education Stress Inventory (HESI-Br) eight-factor exploratory factor analysis (EFA) results (n = 511)

	Factor 1	Factor 2	Factor 3	Factor 4	Factor 5	Factor 6	Factor 7	Factor 8
Proportion explained	0.16	0.16	0.17	0.13	0.10	0.12	0.09	0.07
Q17	0.84	-	-	-	-	-	-	-
Q10	0.73	-	-	-	-	-	-	-
Q26	0.42	0.32	-	-	-	-	-	-
Q6	0.36	-	-	-	-	-	-	-
Q19	0.34	-	-	-	-	-	-	-
Q1	-	-	-	-	-	-	-	-
Q22	-	-	-	-	-	-	-	-
Q8	-	0.80	-	-	-	-	-	-
Q33	-	0.60	-	-	-	-	-	-
Q2	-	0.51	-	-	-	-	-	-
Q27	-	0.43	-	-	-	-	-	-
Q13	-	-	-	-	-	-	-	-
Q31	-	-	0.68	-	-	-	-	-
Q30	-	-	0.55	-	-	-	-	-
Q29	-	-	0.50	-	-	-	-	-
Q32	-	-	0.43	-	-	-	-	-
Q16	-	-	0.38	-	-	-	-	-
Q25	-	-	-	-	-	-	-	-
Q21	-	-	-	-	-	-	-	-
Q12	-	-	-	0.74	-	-	-	-
Q23	-	-	-	0.61	-	-	-	-
Q28	-	-	-	0.57	-	-	-	-
Q20	-	-	-	-	0.61	-	-	-
Q14	-	-	-	-	0.55	-	-	-
Q9	-	-	-	-	-	0.52	-	-
Q11	-	-	-	-	-	0.52	-	-
Q15	-	-	-	-	-	0.39	-	-
Q7	0.37	-	-	-	-	0.37	-	-
Q18	-	-	-	-	-	-	-	-
Q4	-	-	-	-	-	-	0.67	-
Q5	-	0.34	-	-	-	-	0.39	-
Q3	-	-	-	-	-	-	-	-
Q24	-	-	-	-	-	-	-	0.89

**Correlations**	**Factor 1**	**Factor 2**	**Factor 3**	**Factor 4**	**Factor 5**	**Factor 6**	**Factor 7**	**Factor 8**

Factor 1	1.00	-	-	-	-	-	-	-
Factor 2	0.32	1.00	-	-	-	-	-	-
Factor 3	0.15	0.36	1.00	-	-	-	-	-
Factor 4	0.14	0.15	0.42	1.00	-	-	-	-
Factor 5	0.09	-0.01	0.22	0.24	1.00	-	-	-
Factor 6	0.24	0.31	0.44	0.34	0.12	1.00	-	-
Factor 7	0.13	0.25	0.27	0.20	0.15	0.21	1.00	-
Factor 8	0.08	0.11	0.22	0.31	0.05	0.22	0.08	1.00

Factor loadings < 0.3 are not shown.

### Confirmatory factor analysis (CFA)

The analysis confirmed the HESI-Br structure with five factors and 15 items (RMSEA = 0.056, 90% confidence interval [90%CI] 0.047-0.066; CFI = 0.97; TLI = 0.967; SRMR = 0.064). The second-order “Educational Stress” model presented worse fit indices (RMSEA = 0.069, 90%CI 0.060-0.077; CFI = 0.960; TLI = 0.950; SRMR = 0.077) in comparison (p-value < 0.001) with the five-correlated factor model, suggesting multidimensionality. All five factors presented acceptable internal reliabilities, with Cronbach’s α ≥ 0.72 and McDonald’s ω ≥ 0.64. Table 3 contains the CFA results. The analysis of covariance suggests low to moderate correlation between factors (coefficients range from 0.13 to 0.66).

**Table 3:** HESI-Br five-factor model CFA results, item difficulty, and item discrimination parameters (n = 510)

			Item difficulty (parameter β, in z-score)			
				
Factor	Item	Factor loadings (λ)	Totally disagree (1) → Somewhat disagree (2)	Somewhat disagree (2) → Somewhat agree (3)	Somewhat agree (3) → Totally agree (4)	Item discrimination (parameter α)
“Career dissatisfaction” (α = 0.73; ω = 0.75)	10. Not satisfied with choice of career	0.864	0.030	1.118	1.831	3.486
	17. Not proud of profession	0.852	0.142	1.384	2.013	3.271
	6. Personal development not stimulated through studies	0.565	0.339	3.577	5.902	0.672
“Faculty shortcomings” (α = 0.77; ω = 0.74)	8. Lack of encouragement from teachers	0.849	-0.675	0.620	1.692	3.052
	2. Lack of respectful treatment from teachers	0.839	0.385	1.521	2.444	2.349
	33. Lack of feedback from teachers	0.523	-1.558	0.431	2.135	1.235
“Excessive workload” (α = 0.74; ω = 0.70)	29. Too much student-controlled group-activities, resulting in unclear curriculum	0.753	-1.405	0.293	2.396	1.184
	30. Literature too difficult and extensive	0.709	-1.092	-0.019	1.399	2.221
	31. Pace of studies too high	0.664	-1.455	-0.266	1.172	1.918
“Financial concerns” (α = 0.71; ω = 0.64)	12. Worries over financing during education	0.796	-0.953	-0.346	0.730	1.665
	23. Worries about housing	0.666	-0.283	0.272	1.303	1.584
	28. Worries over future economy (debts from studies)	0.552	0.319	0.815	1.857	1.361
“Toxic learning environment” (α = 0.73; ω = 0.68)	15. No acceptance towards weakness and personal shortcomings	0.768	-0.847	0.252	1.420	1.703
	11. Cold and impersonal attitudes enhanced by education	0.693	-0.734	0.602	2.259	1.716
	9. Competitive attitudes among students	0.601	-1.284	-0.209	1.262	1.465

CFA = confirmatory factor analysis; HESI-Br = Brazilian Portuguese version of the Higher Education Stress Inventory; α = Cronbach’s α;

ω = McDonald’s ω.

### Item response theory (IRT) analysis

**Figure 1:**
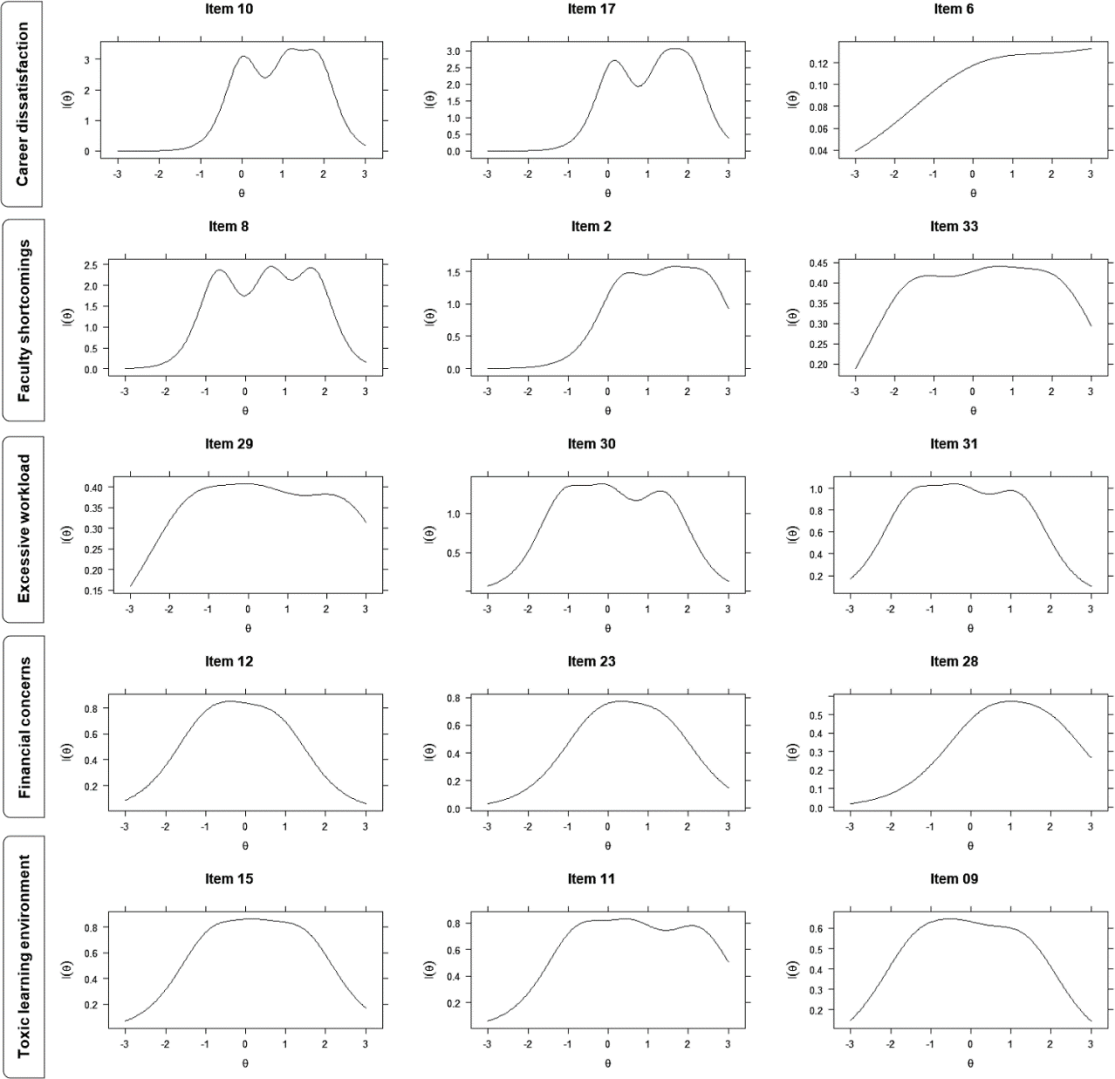
Item information curves for the Brazilian Portuguese version of the HESI (HESI-Br). I(θ) = item information in which the apex of the curve corresponds to the difficulty parameter (β); (θ) = standardized latent construct.

IIC demonstrates that the “career dissatisfaction” factor predominantly captures information on those subjects at the higher end of the stress spectrum (Figure 1). Similarly, most items in the “faculty shortcomings,” “excessive workload,” “financial concerns,” and “toxic learning environment” factors capture information on subjects across the whole spectrum (i.e., -2 to 2 standard deviations [SD] of the latent construct), which indicate that they might be good for screening educational-related stress. Table 3 contains item difficulty and discrimination results for all HESI-Br items. As an example, a person with +0.602 SD of the “toxic learning environment” academic stress construct, has a 50% probability of answering “somewhat disagree” to “somewhat agree” to the “Cold and impersonal attitudes enhanced by education” item. These properties are illustrated in the ICC curves, which reveal that most HESI-Br item response categories are informative for increasing levels of stress (Figure S4, available as online-only supplementary material).

### Measurement equivalence

Measurement equivalence analysis resulted in ΔCFI < 0.01 and ΔRMSEA < 0.015 or ΔSRMR < 0.010 between nested models with increasing levels of constraints. Results are provided in Table 4. It suggests that HESI-Br is equivalent across groups selected by sex, age, study level, family income, and area of study and, therefore, mean education-related stress levels can be compared between these groups.

**Table 4:** HESI-Br measurement equivalence testing

Sample in each group (n)	n	Constraint	RMSEA	CFI	SRMR	Model comparison	Δ RMSEA	Δ CFI	Δ SRMR	Decision
Sex		Configural	0.051	0.980	0.060					
Male	235	Threshold	0.049	0.981	0.060	Configural	0.002	0.001	0.000	Invariant
Female	783	Metric	0.048	0.980	0.061	Threshold	0.001	0.001	0.001	Invariant
		Scalar	0.048	0.979	0.061	Metric	0.000	0.001	0.000	Invariant
Age groups (years)		Configural	0.044	0.985	0.062					
18-25	532	Threshold	0.040	0.986	0.062	Configural	0.004	0.001	0.000	Invariant
26-39	360	Metric	0.039	0.986	0.063	Threshold	0.001	0.000	0.001	Invariant
40+	126	Scalar	0.040	0.984	0.063	Metric	0.001	0.002	0.000	Invariant
Study level		Configural	0.047	0.983	0.064					
Bachelor	619	Threshold	0.043	0.984	0.064	Configural	0.004	0.001	0.000	Invariant
Residency, specialization	132	Metric	0.042	0.984	0.065	Threshold	0.001	0.000	0.001	Invariant
Masters, PhD, post-doc	267	Scalar	0.047	0.978	0.066	Metric	0.005	0.006	0.001	Invariant
Family income		Configural	0.047	0.983	0.065					
Low	223	Threshold	0.043	0.985	0.065	Configural	0.004	0.002	0.000	Invariant
Middle	502	Metric	0.042	0.984	0.066	Threshold	0.001	0.001	0.001	Invariant
High	292	Scalar	0.040	0.984	0.066	Metric	0.002	0.000	0.000	Invariant
Area of study		Configural	0.050	0.982	0.066					
Exact sciences, technology	250	Threshold	0.047	0.982	0.066	Configural	0.003	0.000	0.000	Invariant
Health	379	Metric	0.047	0.980	0.067	Threshold	0.000	0.002	0.001	Invariant
Social, education, arts	380	Scalar	0.047	0.979	0.067	Metric	0.000	0.001	0.000	Invariant

CFI = comparative fit index; HESI-Br = Brazilian Portuguese version of the Higher Education Stress Inventory; RMSEA = root mean square error of approximation; SRMR = standardized root mean-square residual;

Δ = differences between fit indices.

Decision is based on ΔCFI < 0.01 and ΔRMSEA < 0.015 or ΔSRMR < 0.010, which indicate model equivalence.

### Convergent/discriminant validity analysis against Depression, Anxiety and Stress Scale (DASS-21)

The HESI-Br showed discriminant validity in relation to the DASS-21. The second-order factor model presented significantly poorer fit indices (RMSEA = 0.069, 90%CI 0.067-0.071; CFI = 0.983; TLI = 0.982; SRMR = 0.074) in comparison with the model in which DASS-21 and HESI-Br were modeled as two different, but correlated constructs (RMSEA = 0.040, 90%CI 0.037-0.042; CFI = 0.994; TLI = 0.994; SRMR = 0.047). Covariances between HESI-Br factors and the general DASS-21 “distress” factor were low to moderate (coefficients range: 0.19-0.42) and are shown in Table S2, available as online-only supplementary material.

## Discussion

The present study aimed to translate and adapt the HESI to Brazilian Portuguese and evaluate its structure, internal reliability, convergent/discriminant validity, and measurement equivalence. The results indicate that a model with five-correlated factors (career dissatisfaction, toxic environment, faculty shortcomings, excessive workload, and financial concerns) and 15 items is the most suitable structure for the HESI-Br. The five factors presented low to acceptable reliability indices. The highest indices were revealed for “career dissatisfaction,” “faculty shortcomings,” and “excessive workload,” meaning that for those factors sum scores tend to point to the same cohesive construct.

Compared to the seven-factor model with 24 items from the original HESI,^7^ psychometric analysis of the HESI-Br identified a structure with fewer factors and items. However, the identified factors were similar to five of the seven factors described in the original scale (namely, financial concerns, workload, faculty shortcomings, low commitment, and non-supportive climate). The HESI has already been adapted for the Korean and Arabic languages, being validated in medical and nursing students, respectively. Regarding psychometric properties, the K-HESI (Korean version) found a 22-item seven-factor model.^8^ whereas the Arabic-HESI study resulted in a 16-item two-factor model.^15^ It is important to highlight that these differences might have emerged due to methodological factors. For example, items with cross-loadings were eliminated for the Korean HESI and HESI-Br, but not for the Arabic HESI. Beyond this hypothesis, the instrument may have different structures between these countries because the HESI may be non-equivalent, given that educational systems and cultural aspects could be significantly different. Thus, future studies should determine the cross-cultural invariance of the HESI.

The IRT analysis showed that, overall, the instrument captures information about stress on students in the mean levels of the latent academic stressors. However, some items are better for discriminating those with high levels of stress and do not detect those with mild levels of academic stress, such as items 10, 17, 6, 2, and 28. This indicates that while the “Career dissatisfaction” construct (composed by items 10, 17, and 6) is suitable for detection of academic stress in those with high levels of academic-related stress, the other four constructs are better for use for screening purposes.

Furthermore, the HESI-Br may be useful for comparing mean levels of stress among students with different characteristics. Hence, to our knowledge, this is the first study to ascertain measurement equivalence for multiple sample characteristics for the HESI. The HESI-Br showed measurement equivalence on all tested levels (sex, age, education level, area of study, and family income) and, therefore, comparisons among these groups are likely to measure true mean differences in psychological stress. It should be noted, however, that as other versions of the HESI have found different structures, the scale is potentially non-equivalent across countries, as mentioned above.

In the present study, DASS-21 scores had higher correlations with the “financial concerns” factor than with the other HESI-Br factors. Indeed, prevalent economic problems, lack of investment in education, and great inequalities between public and private universities may influence educational stress among students in Brazil. This effect has been captured in a previous meta-analysis, showing that lower family income was associated with higher stress in Brazilian medical students.^5^ Beyond correlation, we analyzed whether the DASS-21 and HESI-Br scales both measured the same latent construct (a general distress factor), but the model fit was inferior to the model in which DASS-21 and HESI-Br were estimated as correlated but structurally different constructs. This suggests academic stress has different characteristics from the distress symptoms that are measured by the DASS-21. This is somewhat expected, since the HESI is intended to measure issues related to stress reported in interviews with students,^7^ but does not capture symptoms of specific disorders.

Some of the HESI-Br factors presented higher correlation with DASS score than others. As seen in Table S2, toxic learning environment and high workload were more strongly correlated with higher general distress than career dissatisfaction and faculty shortcomings. In the original HESI, however, the “low commitment” factor, which is similar to the HESI-Br “career dissatisfaction” factor, showed the highest odds ratio for depressive symptoms measured by the Major Depression Inventory (MDI). Although depression as measured by the MDI in the original HESI study might not translate perfectly when comparing to DASS scores, depressive symptoms are components of the DASS-21,^7^ so it is reasonable to assume some comparability. This comparison highlights potential non-equivalence in student stress between culturally and socioeconomically different countries such as Brazil and Sweden. In Brazil, stress in higher education might be affected by low aspirations tied to uncertain prospects regarding one’s career, which can influence the way that a given student understands items regarding that construct and, therefore, endorses items in a different way. In other words, in high-income countries, lower expectations about one’s career might be more indicative of general distress, whereas in low and middle-income countries, these expectations might already be low, so a high perception of workload and the perception of a toxic learning environment might be more useful for identifying students under significant levels of general distress.

### Strengths and limitations

This study has a number of strengths: (a) a large sample (1,021 students); with (b) students from diverse levels of higher education (undergraduate, graduate, postgraduate); and (c) fields of study. The HESI-Br was invariant in several aspects and can be used in various student populations.

This study has at least four important limitations. First, it used a non-probabilistic sample, selected for convenience from an internet-based survey. However, most of the participants were female, self-declared white, and had high or middle income, which partially reflects the characteristics of the university student population. Second, it is noteworthy that the last phase of validation was conducted within a broad research project, designed with the objective of monitoring the mental health of the Brazilian population during the current pandemic. During the COVID-19 pandemic in Brazil, educational stress among students tends to be even greater, due to education-related stressors, such as distance learning and uncertainties regarding the quality of academic education,^42^ as well as to stressful events external to the student environment, such as financial losses and social isolation.^43^ Future studies should investigate the psychometric properties of the scale when stressors related to the COVID-19 pandemic are not present. Third, the present study does not enable examination of why the structure was different from the other versions. Fourth, the “financial concerns” factor is limited in terms of reliability, but still comprises the best structure for the scale.

## Conclusion

The HESI-Br scale contains 15 items within five factors, namely career dissatisfaction, faculty shortcomings, high workload, financial concerns, and toxic learning environment. The results suggest measurement equivalence by sex, age, educational levels, fields of study, and family income, which indicates comparability of HESI-Br results between groups with different sociodemographic characteristics. Furthermore, IRT analysis suggests the instrument is a potential tool for screening Brazilian university students and can also discriminate those with moderate-to-high levels of stress. Further studies of the HESI-Br should investigate whether it is comparable in different countries and cultures, with different educational systems. Nonetheless, the HESI-Br is a valid tool for screening and assessment of relevant stressors related to higher education in Brazil.
