# Supplementary material for: Translation, cultural adaptation, and validation of the Brazilian Portuguese version of the Higher Education Stress Inventory (HESI-Br)
Source: Trends Psychiatry Psychother. 2023 Sep 22;45:e20210445. doi: 10.47626/2237-6089-2021-0445 (PMC10597388; doi:10.47626/2237-6089-2021-0445)
Supplement: Supplementary file 1 [file 2238-0019-trends-45-e20210445-suppl01.pdf]

http://doi.org/10.47626/2237-6089-2021-0445

Supplementary Material S1

Inventário de Estresse na Educação Superior (IEES)
Versão traduzida para o Português (Brasil)
Higher Education Stress Inventory (HESI)

Abaixo você encontrará diversas afirmações sobre sua relação e experiência pessoal com o ambiente acadêmico. Por favor, para cada uma delas, indique o seu grau de concordância.
Os termos ‘estudo’ e ‘estudos’ se referem a todas as suas atividades acadêmicas e carga de trabalho.

**Inventário de Estresse na Educação Superior (IEES) – IEES – tabela de pontuação**		Discordo totalmente	Discordo parcialmente	Concordo parcialmente	Concordo totalmente
1	Os estudos controlam a minha vida e tenho pouco tempo para outras atividades.	1	2	3	4
2	Eu sinto que meus professores me tratam com respeito.	4	3	2	1
3	Eu me preocupo de que não vou adquirir todos os conhecimentos necessários para minha futura profissão.	1	2	3	4
4	Os estudos criaram isolamento e desconhecimento entre os estudantes.	1	2	3	4
5	Os professores frequentemente falham em esclarecer o objetivo dos estudos.	1	2	3	4
6	Os estudos estimulam meu desenvolvimento pessoal.	4	3	2	1
7	O papel profissional apresentado em minha formação entra em conflito com minhas visões pessoais.	1	2	3	4
8	Os professores dão incentivo e atenção pessoal.	4	3	2	1
9	Existe uma atitude competitiva entre os estudantes.	1	2	3	4
10	Eu estou satisfeito(a) com a escolha da minha carreira.	4	3	2	1
11	Eu sinto que os estudos tiveram um papel na criação de uma atitude fria e impessoal entre os estudantes.	1	2	3	4
12	Como estudante, minha situação financeira é uma preocupação.	1	2	3	4
13	Meus colegas estudantes me dão apoio.	4	3	2	1
14	Eu me preocupo com longas jornadas de trabalho e com as responsabilidades da minha futura carreira.	1	2	3	4
15	Meu treinamento é caracterizado por uma atmosfera em que fraquezas e imperfeições pessoais não são aceitas.	1	2	3	4
16	Como estudante, é frequentemente esperado que eu participe de situações em que meu papel e função não são claros.	1	2	3	4
17	Estou orgulhoso(a) de minha profissão futura.	4	3	2	1
18	Sinto que sou tratado(a) de forma pior por causa de meu gênero.	4	3	2	1
19	Eu posso exercer influência sobre o meu currículo (incluindo atividades optativas e extracurriculares).	1	2	3	4

20	O *insight* (percepção) que tenho tido da minha futura profissão tem me causado preocupação sobre uma carga de trabalho estressante.	1	2	3	4
21	Existe demasiado foco no aprendizado passivo dos fatos e muito pouco em busca ativa de conhecimento e tempo para reflexão.	1	2	3	4
22	Expectativas da minha família influenciaram demais a escolha de minha carreira.	1	2	3	4
23	Eu estou preocupado(a) com questões relacionadas a moradia (habitação, local para morar).	1	2	3	4
24	Eu sinto que sou tratado(a) de forma pior por causa da minha origem étnica.	1	2	3	4
25	Conheço vários futuros colegas que parecem abatidos ou insatisfeitos em sua profissão.	1	2	3	4
26	Eu sinto que minha formação está me preparando bem para minha futura profissão.	4	3	2	1
27	As atividades do centro/diretório acadêmico promovem um senso de comunidade e contribuem para um melhor ambiente de trabalho para os estudantes.	4	3	2	1
28	Estou preocupado(a) com minhas economias futuras e minha habilidade em pagar o financiamento estudantil.	1	2	3	4
29	A educação é altamente caracterizada por atividades em grupo. Consequentemente, os objetivos são pouco claros e responsabilidade demais é colocada no estudante.	1	2	3	4
30	A literatura é demasiadamente difícil e extensa.	1	2	3	4
31	O ritmo de estudos é acelerado demais.	1	2	3	4
32	A formação demanda que eu participe de situações que eu acho antiéticas.	1	2	3	4
33	Os professores frequentemente dão retorno sobre o conhecimento e habilidades dos estudantes.	4	3	2	1

Estudo original: Dahlin M, Joneborg N, Runeson B. Stress and depression among medical students: a cross-sectional study. Medical Education 2005;39(6):594-604.

Table S1 - Response frequency (%) of Higher Education Stress Inventory (HESI) items (n = 1,021)

	Totally disagree	Somewhat disagree	Somewhat agree	Totally agree
Q1	0.21	0.23	0.40	0.16
Q2*	0.02	0.07	0.28	0.63
Q3	0.12	0.12	0.36	0.39
Q4	0.21	0.28	0.36	0.15
Q5	0.27	0.34	0.31	0.08
Q6*	0.02	0.06	0.34	0.57
Q7	0.51	0.27	0.18	0.04
Q8*	0.06	0.22	0.46	0.27
Q9	0.22	0.24	0.33	0.21
Q10*	0.04	0.10	0.34	0.53
Q11	0.31	0.34	0.29	0.06
Q12	0.25	0.17	0.29	0.29
Q13*	0.10	0.15	0.49	0.26
Q14	0.11	0.13	0.34	0.41
Q15	0.29	0.28	0.27	0.16
Q16	0.23	0.27	0.39	0.11
Q17*	0.03	0.07	0.33	0.57
Q18	0.53	0.20	0.19	0.08
Q19*	0.06	0.11	0.39	0.44
Q20	0.18	0.22	0.37	0.23
Q21	0.16	0.28	0.41	0.16
Q22	0.49	0.22	0.20	0.09
Q23	0.44	0.16	0.24	0.17
Q24	0.89	0.05	0.05	0.01
Q25	0.19	0.22	0.38	0.20
Q26*	0.05	0.19	0.52	0.23
Q27*	0.24	0.23	0.40	0.14
Q28	0.58	0.13	0.18	0.11
Q29	0.22	0.37	0.33	0.08
Q30	0.18	0.31	0.37	0.14
Q31	0.15	0.27	0.38	0.20
Q32	0.64	0.22	0.11	0.02
Q33*	0.12	0.28	0.42	0.18

* Reverse-coded.

Table S2 - Correlations between the 5 factors of the Brazilian Portuguese version of the HESI (HESI-Br) and with the Depression, Anxiety and Stress Scale (DASS-21) general distress factor

Factor	Career dissatisfaction	Faculty shortcomings	High workload	Financial concerns	Toxic learning environment
Career dissatisfaction	1.00	-	-	-	-
Faculty shortcomings	0.461	1.00	-	-	-
High workload	0.195	0.384	1.00	-	-
Financial concerns	0.209	0.217	0.459	1.00	-
Toxic learning environment	0.357	0.524	0.631	0.473	1.00
Distress	0.296	0.193	0.368	0.424	0.389

The DASS-21 general distress factor is the higher-order factor representing the correlation between depression, anxiety, and stress first-order factors.

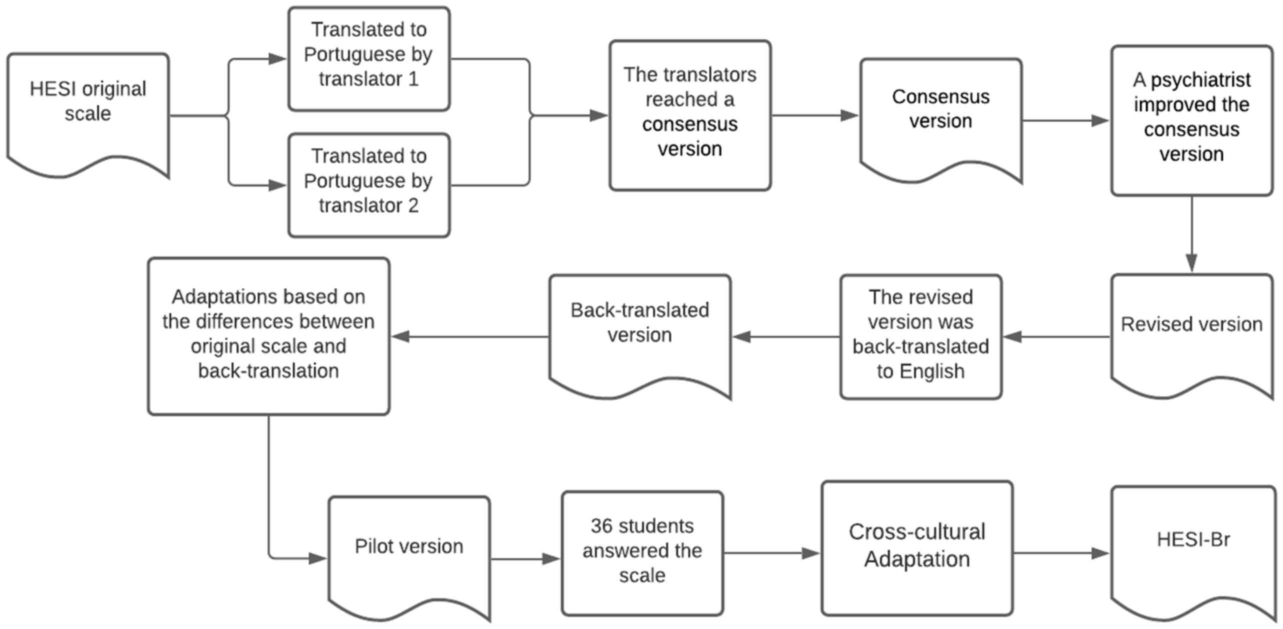

Figure S1 - Flowchart illustrating translation and cross-cultural adaptation of the Brazilian Portuguese version of the HESI (HESI-Br). A perfect rectangle represents a process. A rectangle with a curved side represents a document.

http://doi.org/10.47626/2237-6089-2021-0445

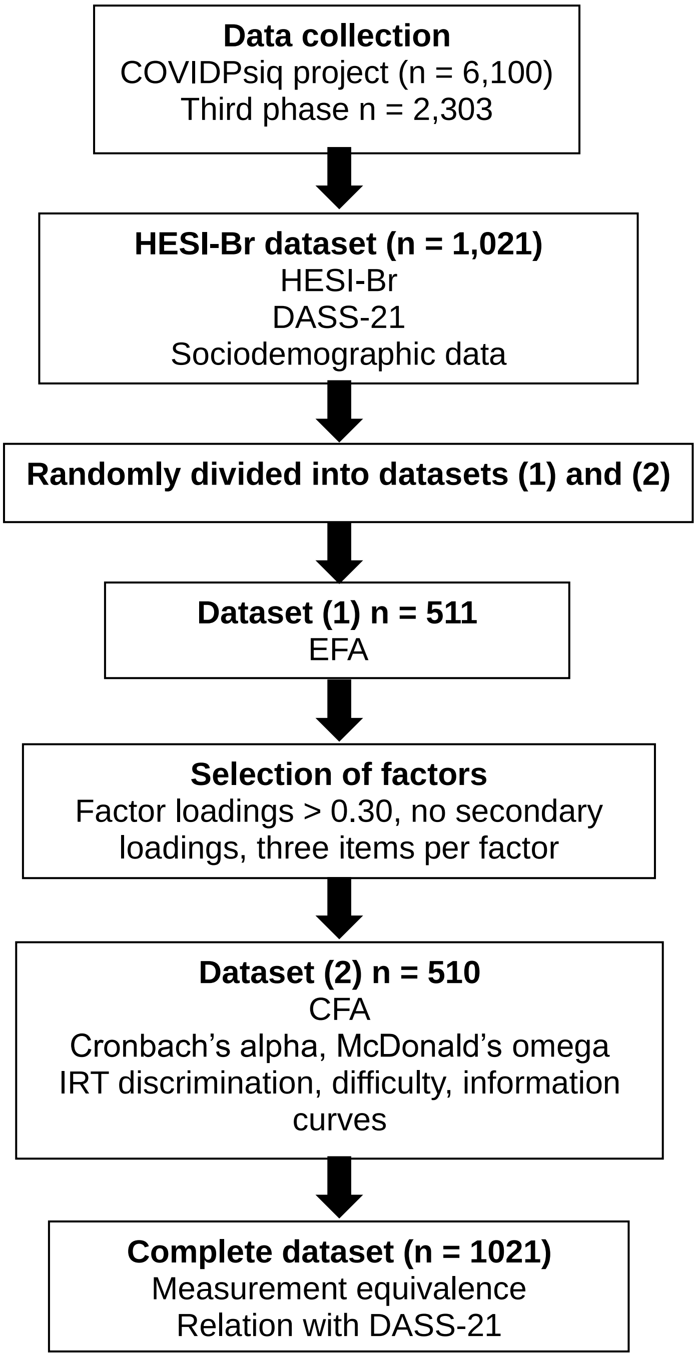

Figure S2 - Data analysis flowchart. CFA = confirmatory factor analysis; DASS-21 = Depression, Anxiety and Stress Scale; EFA = exploratory factor analysis; HESI-Br = Brazilian Portuguese version of the Higher Education Stress Inventory; IRT = item response theory.

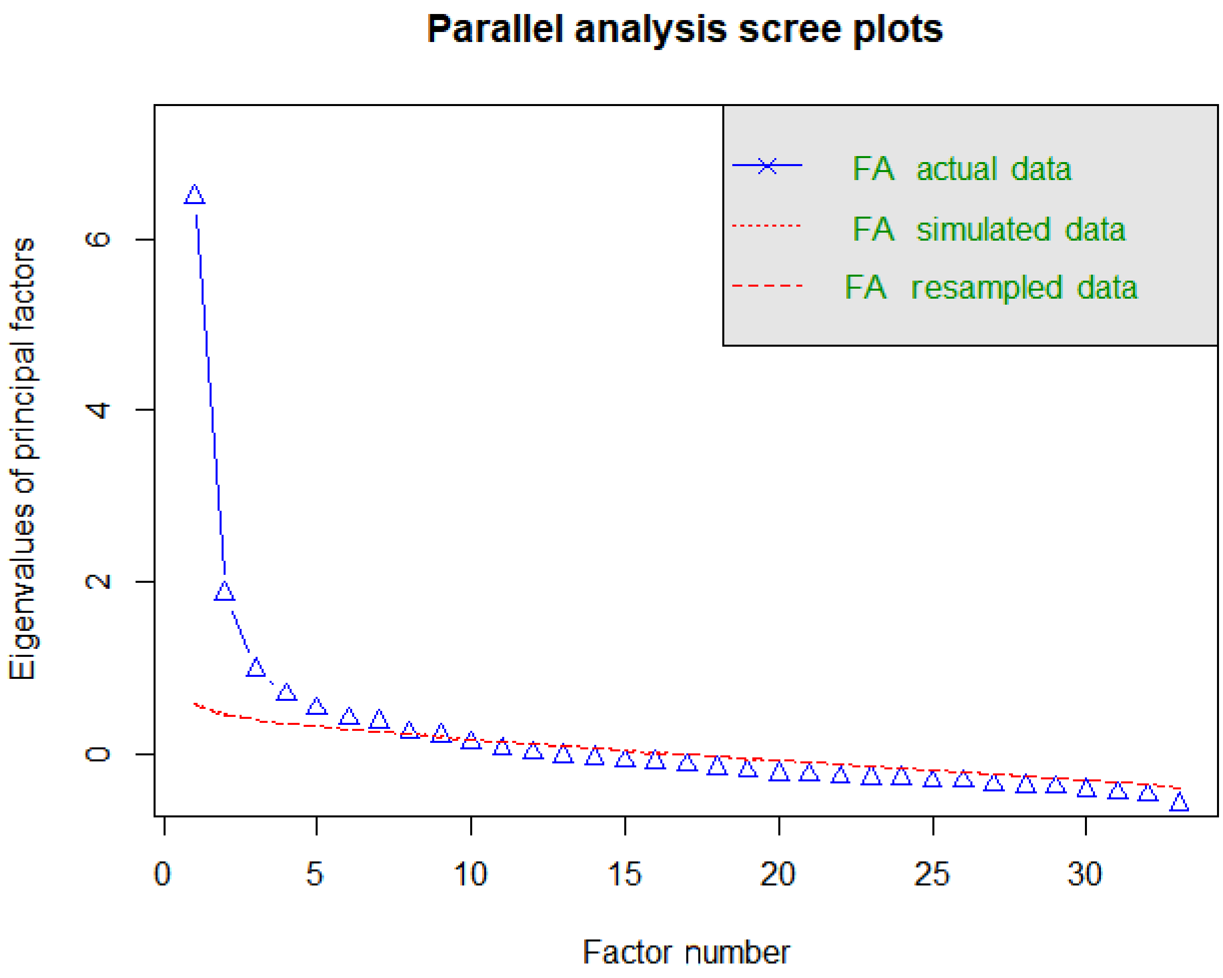

Figure S3 - Scree plot of parallel analysis using weighted least squares for the Brazilian Portuguese version of the HESI (HESI-Br). FA = factor analysis.

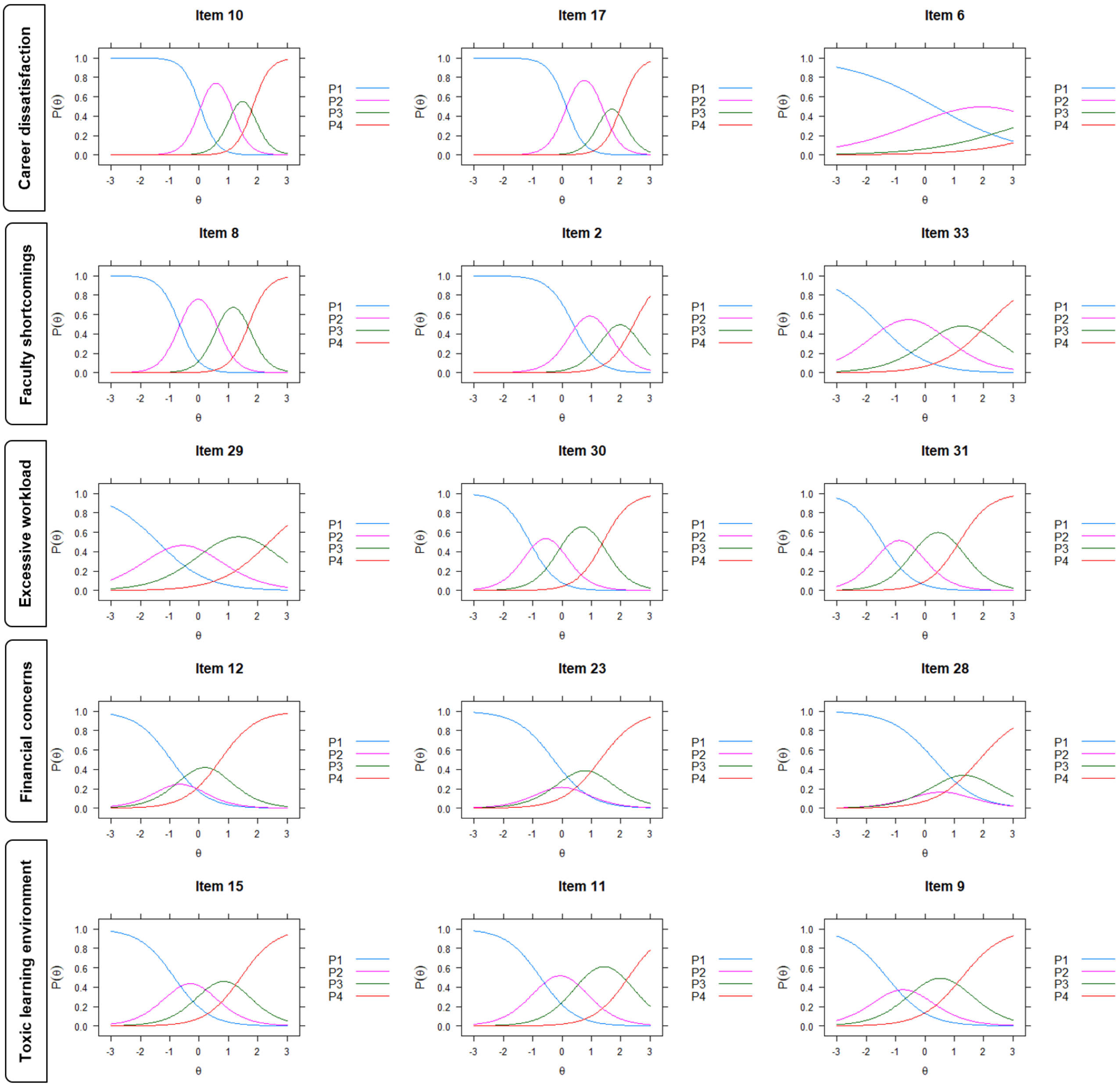

Figure S4 - Item characteristic curves for the Brazilian Portuguese version of the HESI (HESI-Br). P(θ) = answer probability; θ = standardized latent construct.
